# Increased prediction accuracy using a genomic feature model including prior information on quantitative trait locus regions in purebred Danish Duroc pigs

**DOI:** 10.1186/s12863-015-0322-9

**Published:** 2016-01-05

**Authors:** Pernille Sarup, Just Jensen, Tage Ostersen, Mark Henryon, Peter Sørensen

**Affiliations:** Department of Molecular Biology and Genetics, Center for Quantitative Genetics and Genomics, Aarhus University, Blichers Allé 20, 8830 Tjele, Denmark; SEGES Danish Pig Research Centre, Axeltorv 3, 1609 Copenhagen V, Denmark

**Keywords:** Genomic feature models, GFBLUP, Feed efficiency, Average daily gain, Meat percent, Growth, Genomic prediction

## Abstract

**Background:**

In animal breeding, genetic variance for complex traits is often estimated using linear mixed models that incorporate information from single nucleotide polymorphism (SNP) markers using a realized genomic relationship matrix. In such models, individual genetic markers are weighted equally and genomic variation is treated as a “black box.” This approach is useful for selecting animals with high genetic potential, but it does not generate or utilise knowledge of the biological mechanisms underlying trait variation. Here we propose a linear mixed-model approach that can evaluate the collective effects of sets of SNPs and thereby open the “black box.” The described genomic feature best linear unbiased prediction (GFBLUP) model has two components that are defined by genomic features.

**Results:**

We analysed data on average daily gain, feed efficiency, and lean meat percentage from 3,085 Duroc boars, along with genotypes from a 60 K SNP chip. In addition information on known quantitative trait loci (QTL) from the animal QTL database was integrated in the GFBLUP as a genomic feature. Our results showed that the most significant QTL categories were indeed biologically meaningful. Additionally, for high heritability traits, prediction accuracy was improved by the incorporation of biological knowledge in prediction models. A simulation study using the real genotypes and simulated phenotypes demonstrated challenges regarding detection of causal variants in low to medium heritability traits.

**Conclusions:**

The GFBLUP model showed increased predictive ability when enough causal variants were included in the genomic feature to explain over 10 % of the genomic variance, and when dilution by non-causal markers was minimal. In the observed data set, predictive ability was increased by the inclusion of prior QTL information obtained outside the training data set, but only for the trait with highest heritability.

**Electronic supplementary material:**

The online version of this article (doi:10.1186/s12863-015-0322-9) contains supplementary material, which is available to authorized users.

## Background

Standard genomic best linear unbiased prediction (GBLUP) models produce accurate predictions of genetic merit when applied in highly structured populations with many close relationships, as typically found in livestock species [1]. GBLUP models infer genetic relationships from genetic markers, which are used to construct a realized genomic relationship matrix [2]. In populations with a high degree of linkage disequilibrium, the determined genomic relationships may provide accurate information about the underlying causal genetic variation [3]. The genomic relationship matrix can be constructed in several different ways. Often the individual genetic markers contribute equally to the genomic relationships (perhaps weighted according to minor allele frequencies) [4]. As a result, genomic variation is generally treated as a “black box,” ignoring any available information regarding functional features of the genome.

However, genome-wide association studies suggest that many genetic variants with independent effects are located in the same genes, and that many of these genes are connected via biological pathways [5]. Thus, extensions of the standard GBLUP modelling approach have been proposed to incorporate available information regarding causal marker distribution along the genome or biological mechanisms underlying trait variation [6–8]. Such approaches may increase prediction accuracy in populations with low levels of genetic relatedness, but not in populations with highly related individuals (e.g. inbred mice stocks [7]). Further studies are required to determine the factors that influence prediction model accuracy in populations with close relationships, such as purebred pig populations [9]. Additionally, patterns in GBLUP-derived single-marker statistics (e.g. estimates of single-marker additive genetic effects) can reveal associations between a genomic feature and a complex trait [10]. These associations represent novel insights into the genetic mechanisms underlying a trait, and may be used to develop more accurate genomic feature BLUP (GFBLUP) models.

We present a GFBLUP modelling approach in the present paper. We investigated whether its use could increase prediction accuracy using real and simulated phenotypes from a purebred Danish Duroc pig population comprising highly related individuals [9]. The tested GFBLUP model is an extension of the linear mixed model used in standard GBLUP. The novel model includes an additional genetic effect that quantifies the collective action of sets of genetic markers on the trait phenotypes, which can include prior data regarding genomic features, e.g. genomic regions containing previously identified quantitative trait loci (QTL).

Information on known QTL regions is available in several publicly available databases, such as Animal QTLdb [11]. QTLs are genomic regions containing one or more putative causal variants, which may be associated with one or more complex traits in different study populations or breeds, potentially varying in effect size. These regions will also span several non-causal variants. Several properties of known QTLs can influence the predictive ability of the GFBLUP modelling approach and the power to detect which marker sets affect a trait. The first potentially influential factor is the proportion of the total genetic variance in a trait that is explained by known QTLs. The second is the number of non-causal variants included in the QTL regions. Third, the model’s power can be impacted by the genetic architecture of QTLs, e.g. whether the causal variants are distributed randomly or clustered along the genome. Furthermore, the model may be affected by population and trait-specific factors, e.g. the total heritability of a trait and the number of observations available for analysis.

Here we applied our GFBLUP approach to analyse growth rate, feed efficiency, and lean meat percentage in pure-bred Danish Duroc boars (*Sus scrofa*) using genomic features defined by the QTL categories listed in the Pig QTLdb database [11]. To attain insight into the biological mechanisms causing trait variation, we identified genomic features that were enriched for associated SNPs. We further investigated the usefulness of this information in a population with highly related individuals by comparing the predictive ability of linear mixed model approaches that either utilised or ignored prior information regarding known QTL regions. Furthermore, we simulated phenotypes based on the observed genotypes of the Danish Duroc population, in order to understand the impact of the above-mentioned five QTL-, population-, or trait-specific factors on the predictive ability of GFBLUP modelling approaches in a population with strong family relationships.

The aims of this study included evaluating the GFBLUP modelling approach by identifying properties of the previously identified QTL regions that influence prediction accuracy. We also tested the GFBLUP using genomic and phenotypic data from the Danish Duroc population, and to thus provide novel insight into the genetic architecture and biological background of growth phenotypes in pigs. We hypothesized that partitioning genomic variation using GFBLUP would increase predictive ability in a population of highly related individuals, but that this increase would be partly dependent on the power to identify true causal QTLs or significant marker sets.

## Results

### The impact of factors—simulated data sets

The simulated data sets included variations of five factors that potentially affect power, with the aim of detecting marker sets that included causal variants and that affected predictive ability of the GFBLUP model. In all scenarios, the sum of t^2^ (the squared value of the single-marker *t*-test statistic) of the markers in the genomic feature performed as well as or better than the other single-marker test statistics (Additional file 1). Therefore, the results presented below are based on this statistic.

#### Power to detect marker sets with causal variants

**Fig. 1:**
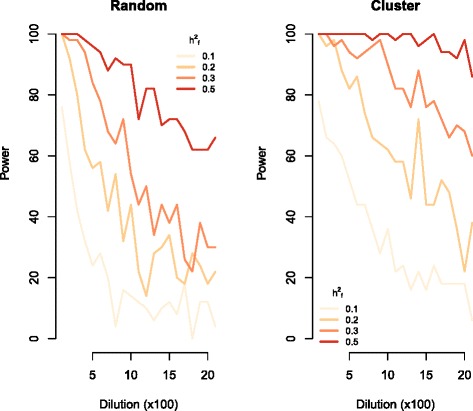
Graphs depict the power to detect marker sets that include true causal variants within a simulated phenotype data set with h^2^ = 0.2 and comprising 3,000 animals, as a function of dilution through the inclusion of non-causal markers in the genomic feature marker set. Each panel shows the effect of varying the fraction of the genetic variance explained by the causal markers in the genomic feature from 0.1 to 0.5. The left panel shows results from random causal models, while the right panel shows the corresponding results from cluster causal models

We investigated the effects of the five different QTL-, population-, or trait-specific factors in terms of the power to detect marker sets including causal variants. In all scenarios, the false positive rate was ≤0.05. Compared to the random causal model, the cluster causal model was more robust to dilution by non-causal SNPs in the marker set (Fig. 1). In the absence of dilution, the two types of genetic models did not differ in power. Below, we present the results from the cluster causal model.

**Fig. 2:**
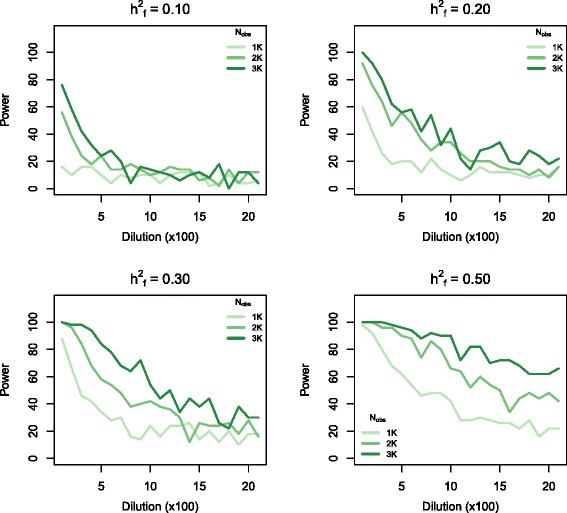
Graphs show the power to detect marker sets that include true causal variants within simulated phenotype data with h^2^ = 0.2, as a function of dilution through inclusion of non-causal markers in the genomic feature marker set. All panels show the effect of varying sample size from 1,000 to 3,000 animals. The four panels each correspond to a different fraction of the genetic variance being explained by the causal markers in the genomic feature (increasing from 0.1 to 0.5). The causal markers were distributed randomly along the genome

**Fig. 3:**
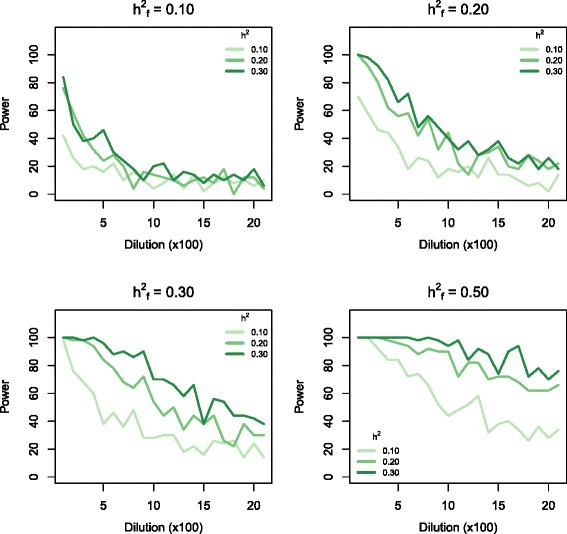
The graphs show the power to detect marker sets that include true causal variants within a simulated phenotype data set comprising 3,000 animals, as a function of dilution through inclusion of non-causal markers in the genomic feature marker set. All panels show the effect of varying h^2^ from 0.1 to 0.3. The four panels each correspond to different fractions of the genetic variance being explained by the causal markers in the genomic feature (increasing from 0.1 to 0.5). The causal markers were distributed randomly along the genome

**Fig. 4:**
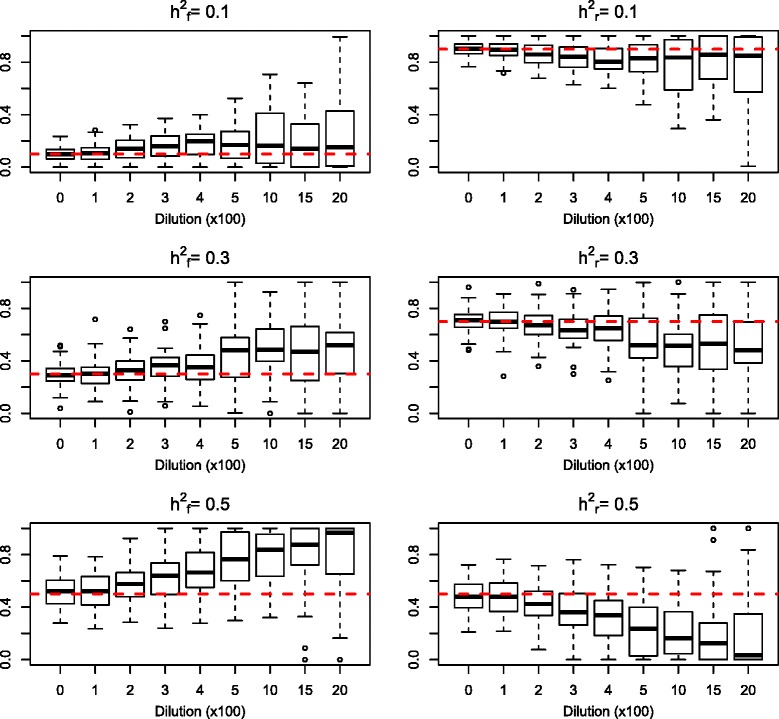
Plots showing the proportions of genetic variance attributed to the genomic feature (left hand column) and to the remaining markers (right hand column). The results shown are from the *cluster model*, and did not differ from the results of the *random model*

In all simulation scenarios, power was decreased by dilution of the effect of causal markers in a marker set by including non-causal markers in the set (Figs. 2, 3. and 4). The proportion of the genomic variance explained by the causal variants included in the genomic feature (h^2^_f_) greatly impacted the detection power (Figs. 2, 3 and 4) and robustness against dilution. At h^2^_f_ = 0.1, no simulation scenario had an average power of >0.8, and there was almost no power to detect marker sets that included causal variants if N_obs_ or h^2^ was low, even without dilution. If the causal variant effect was diluted by including non-causal markers in the marker sets, the power was very low in all simulation scenarios (Figs. 2, 3 and 4). At the highest h^2^_f_, the impact of dilution was much less severe. This increased robustness towards dilution resulted in power of >70 % in all cluster model scenarios with 3 K observations and a heritability of 0.3 (Fig. 3, lower right panel).

We found that power was positively correlated with the number of observations (N_obs_) (Fig. 2). At h^2^_f_ = 0.1, the power with a N_obs_ of 3 K was 4-fold higher than that at 1 K. This difference in power decreased with increasing h^2^_f_ . At h^2^_f_ = 0.5, all scenarios with h^2^ = 0.2 detected all sets that included causal variants, provided that there was no dilution (Fig. 2, lower right panel). Increasing the number of observations increased the robustness towards dilution, especially in simulations with high h^2^_f_. This increased robustness resulted in shallower slopes of the lines representing 2 K and 3 K observations in Fig. 2 (lower right panel). Power was also positively correlated with h^2^ (Fig. 3). However, at high h^2^_f_ and in the absence of dilution, all marker sets including causal variants were detected regardless of overall heritability. In simulations with high h^2^_f_, high heritability traits were less affected by dilution than low heritability traits (Fig. 3, lower half).

#### Partitioning of genomic variance by GFBLUP

In all simulation scenarios, the estimation of total genomic heritability was unbiased, as $\hat{{\mathrm{h}}^2}$ estimated by equation (M_GF_) was equal to the h^2^ used for simulation of the data. Furthermore, the estimation of the proportion of genomic variance that was attributed to the markers associated with the genomic feature (h^2^_f_) was unbiased in scenarios with low dilution by non-causal variants in the genomic feature (Fig. 4). Increased dilution led to increased variance of the estimated ${\hat{\mathrm{h}}}_{\mathrm{f}}^2$. Additionally, in scenarios where the true h^2^_f_ was >0.1, the estimated ${\hat{\mathrm{h}}}_{\mathrm{f}}^2$ was increasingly upward biased with greater dilution.

#### Predictive ability of GFBLUP

**Fig. 5:**
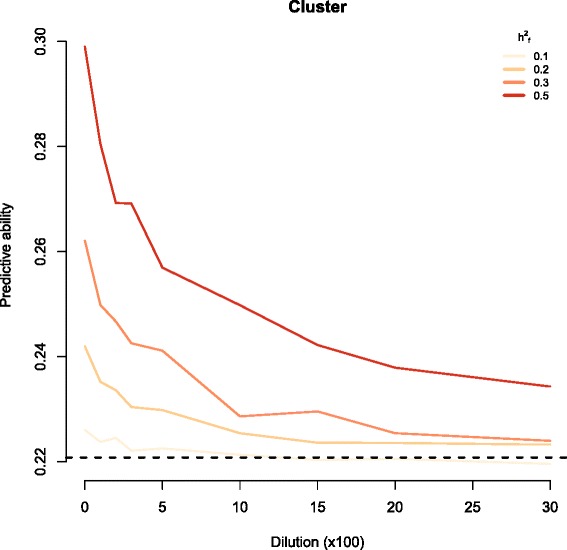
Plot depicting the predictive ability of the simulated phenotype data set with h^2^ = 0.2, as a function of dilution through inclusion of non-causal markers in the genomic feature marker set. The effect of varying the fraction of the genetic variance that is explained by the causal markers in the genomic feature from 0.1 to 0.5 in the cluster causal model is shown

We investigated the effects of dilution and h^2^_f_ on predictive ability when h^2^ was kept constant at 0.20. The design of the validation study was identical to the one used in the real data set. The maximum correlation between the phenotypic observations and the genomic values is the square root of the heritability—in this case h = 0.45. We found a correlation of 0.22 between the observation and the genomic values of the standard GBLUP. The GFBLUP had higher predictive abilities with a correlation of up to 0.30, as long as there was a high proportion of genomic variation caused by the causal markers in the marker set, with few non-causal markers included. Thus, the effects of h^2^_f_ and dilution on predictive ability were similar to their effects on power (Fig. 5). These findings highlight the importance of maximising the proportion of causal variants in **G**_f_. In contrast, predictive ability did not differ between the cluster and random causal variant models (results not shown).

### Comparing genomic models using observed data

Comparing the different genomic model approaches based on their genomic heritability and their predictive ability in the real data set enabled us to evaluate how well the models fitted the data, as well as the utility of the GBLUP and GFBLUP models. Estimates of heritability, $\hat{{\mathrm{h}}^2}$ using equation (M_a_) were 0.36, 0.19, and 0.12 for the lean meat percentage (LMP), feed efficiency (FE), and average daily gain (ADG), respectively. The heritability of the corrected phenotype (used as phenotype for the genomic models) that were explained by the animal effect, $\frac{{\hat{\sigma}}_a^2}{{\hat{\sigma}}_a^2+{\hat{\sigma}}_e^2}$, for LMP, FE, and ADG were 0.42, 0.20, and 0.26, respectively.

#### Comparing genomic heritability and partitioning of genetic variance among genomic models

**Fig. 6:**
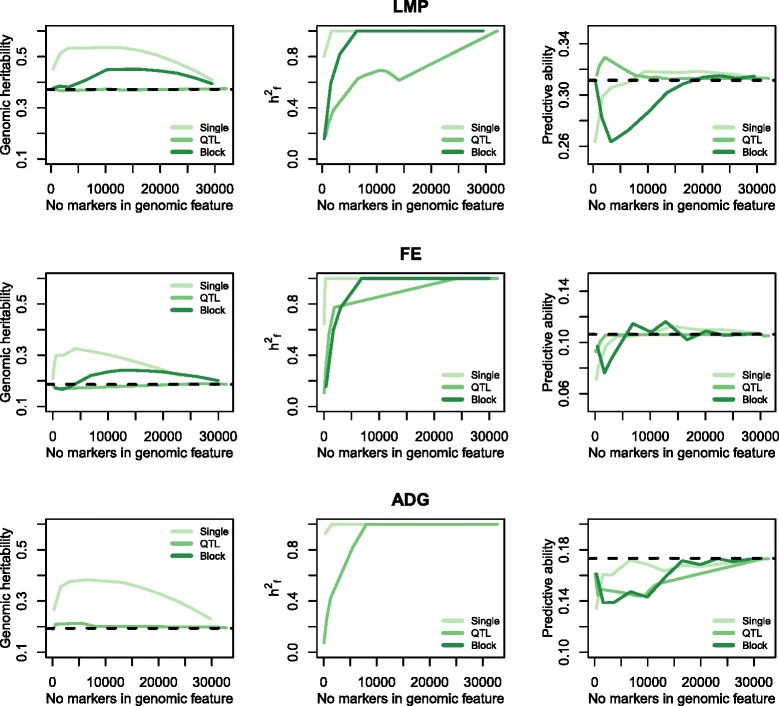
Graphs in the left column show the genomic heritability of GFBLUP for lean meat percentage, feed efficiency, and average daily gain as a function of the number of markers included in the genomic feature. The black dotted line represents genomic heritability of a standard GBLUP. Graphs in the middle column show the proportion of genetic variance explained by the genomic feature as a function of the number of markers included in the genomic feature. Graphs in the right column depict the correlation between the phenotype and the sum of genetic values for the genomic feature and the rest marker sets plotted as a function of the number of markers included in the genomic feature. The black dotted line represents the correlation between the phenotype and genetic values from a standard GBLUP

Estimates of genomic heritability, $\hat{{\mathrm{h}}^2}$ in the training set using equation (M_GF_) differed greatly between the genomic feature classes that did not include information from other sources than our data set, single-marker and block set models, and the QTL set models for all three traits (Fig. 6). QTL set models explained proportions of variance that were similar to the standard GBLUP. However, the genomic heritabilities of the single-marker and block set models were much higher than both the QTL set and the standard GBLUP for all three traits. When there were more than a few hundred SNPs in a genomic feature, almost all of the genomic variance was captured by the genomic feature (Fig. 6). This resulted in the genomic variance of the feature set $\left({\hat{\mathrm{h}}}_{\mathrm{f}}^2\right)$ in all models and traits, except for the QTL set models for LMP. The single-marker set models were most extreme, with only the two lowest *p* value cut-off models showing ${\hat{\mathrm{h}}}_{\mathrm{f}}^2<{\hat{\mathrm{h}}}^2$. For QTL set models for LMP, ${\hat{\mathrm{h}}}_{\mathrm{f}}^2$ increased at a lower rate and then decreased again along with an increasing number of markers in the genomic feature.

#### Comparing predictive ability between genomic models

The last column of Fig. 6 depicts the model predictive ability measured as the correlation between **y** and $$ \hat{\mathbf{g}\ } $$$$ \left(\mathrm{f}\mathrm{o}\mathrm{r}\ \mathrm{GFBLUP}:\ \hat{\mathbf{g}} = {\hat{\mathbf{g}}}_f+{\hat{\mathbf{g}}}_r\right) $$. The predictive ability was significantly improved for LMP in the best-performing QTL set model with a *p* value cut-off of 0.1, showing a 5.6 % increase compared to the standard GBLUP. However, we found no improvement of predictive ability for any GFBLUP model for FE or ADG. Despite the much higher genomic heritability in the training set (Fig. 6), none of the single-marker or block set models using equation (M_GF_) showed higher predictive ability than the standard GBLUP (Fig. 6).

In lieu of the GFBLUP presented in equation (M_GF_), an alternative strategy was to use **G** including all markers as the second component instead of **G**_r_. This alternative GFBLUP approach resulted in the same estimates of genomic heritability and predictive ability as the GFBLUP in equation (M_GF_) (results not shown). We also tested the method presented by Zhang *et al*. [6], in which each marker is weighted according to the number of times its position is reportedly within a QTL. This model showed the same predictive ability as the standard GBLUP (results not shown).

#### QTL sets associated with growth phenotypes

Table 1 list the *p* values for the QTL sets for LMP, FE, and ADG for which at least one *p* value was <0.1. The QTL sets included in **G**_**f**_ in the best-performing GFBLUP for LMP can be grouped into four categories: muscle QTLs, adipose QTL sets, immune system QTLs, and body conformation QTLs.

**Table 1:** QTL sets for which *p* was <0.1 (in bold) for any of the three phenotypes

QTL set	nrSNP	LMP	ADG	FE
Leukocyte quantity	314	**0.0011**	0.2387	**0.0919**
Cannon bone circumference	76	**0.0024**	0.8714	**0.0995**
Head mass	259	**0.0025**	0.5209	0.4896
Testes mass	36	**0.0041**	0.3605	0.8389
White adipose amount	31	**0.0088**	**0.0389**	0.1443
Total foot mass	22	**0.0186**	0.7994	0.3193
CD4-positive T cell quantity	3	**0.0278**	0.6724	0.1976
Blood acidity-alkalinity balance trait	47	**0.0308**	0.1103	0.9526
White adipocyte size trait	284	**0.0346**	0.8514	0.9304
Outer ear area	62	**0.0421**	0.4305	0.1122
Longissimus dorsi muscle thickness	120	**0.0446**	0.9997	0.1073
Type IIa muscle fibre quantity	92	**0.0519**	0.4368	0.9979
Nipple quantity	622	**0.0542**	0.1287	0.8911
Sperm quantity	23	**0.0702**	0.8426	0.8555
Skeletal muscle fibre quantity	51	**0.0732**	0.6365	0.1617
Type IIb muscle fibre quantity	147	**0.0739**	0.5245	0.4762
Blood interleukin-10 amount	60	**0.0797**	0.1755	0.3106
Vertebra quantity	103	**0.0819**	0.6869	0.5845
Thoracic vertebra quantity	52	**0.0890**	0.1276	0.9734
Blood haemoglobin amount	230	**0.0985**	0.7056	0.6039
Bone mineral mass	31	0.1462	**0.0563**	0.9503
Sperm morphology trait	24	0.1523	**0.0235**	0.7538
Blood LDL cholesterol amount	70	0.1594	**0.0078**	0.2317
Locomotor activity trait	24	0.2583	**0.0661**	0.1148
Sperm motility trait	27	0.2895	**0.0384**	0.5715
Muscle water amount	321	0.3356	**0.0366**	0.5561
Lung mass	15	0.3399	0.4898	**0.0422**
Blood bilirubin amount	16	0.3609	**0.0561**	**0.0868**
Erythrocyte size trait	348	0.3836	**0.0617**	0.6727
Glycolytic potential	129	0.3889	0.2145	**0.0438**
Limb conformation trait	11	0.4034	0.9988	**0.0005**
Blood sodium amount	34	0.4449	**0.0465**	0.9931
Blood adrenocorticotropin amount	20	0.4575	**0.0563**	0.1920
Ejaculation trait	25	0.5113	0.4116	**0.0035**
Hindlimb muscle mass	94	0.5439	0.7693	**0.0248**
White adipose mass	110	0.5492	**0.0241**	0.6643
Femur mineral mass	20	0.6386	**0.0449**	0.1339
Skeletal muscle fibre size trait	252	0.6731	0.8899	**0.0847**
Anus morphology trait	141	0.7901	**0.0541**	0.9144
Rump morphology trait	101	0.8217	0.8194	**0.0709**
Skeletal muscle myosin isoform amount	9	0.8224	**0.0919**	0.5332
Blood leptin amount	26	0.8593	0.4058	**0.0783**
Spleen mass	138	0.9181	**0.0551**	0.5360

Each QTL set was tested independently

## Discussion

The analysis of both simulated and real data sets showed that GFBLUP approaches have the potential to increase prediction accuracy in the Danish Duroc population. Whether this potential is realised or not depends upon a number of factors which we will discuss in detail below.

### Investigating the impact of factors using simulated data sets

We investigated the factors that could affect SNP set-based partitioning of genomic variance (Table 2), as well as influence the power to detect significant genomic features within a highly structured data set, such as the Danish Duroc population.

**Table 2:** Summary of simulation factors

Factor	Levels
h^2^	(3) 10 %, 20 %, 30 %
h_f_^2^	(4) 10 %, 20 %, 30 %, 50 %
Dilution	(20) 100, 200, …, 2000
Genome distribution of causal SNPs	(2) Random or Clustered
Number of observations	(3) 1 K, 2 K, 3 K

#### Impact on power to detect marker sets with causal variants

For traits with medium heritability (h^2^ = 0.2), we found power ranging from 0.6 to 1 for the detection of marker sets that included causal variants within a sample size comparable to that of the training data set. The changes in power were related to the proportion of genomic variance explained by the causal marker set, when no non-causal markers were included in **G**_f_ (Fig. 1). Dilution of the causal marker set by addition of non-causal markers (dilution sets) reduced the power. Causal dilution sets could only be detected in scenarios in which all other factors were tuned to maximise power (Figs. 2, 3 and 4). Such scenarios were characterized by high proportions of the total genomic variance being explained by the causal variants included in the marker set (C_1_), and large numbers of observations.

In scenarios where h_f_^2^ was 0.1, each causal SNP in C_1_ explained the same proportion of the genetic variance as the individual SNPs in C_2_ (causal SNPs not included in the marker set). In these scenarios, power was very low when C_1_ was diluted by non-causal variants included in the marker set, regardless of the number of observations and heritability (Fig. 3). Notably, the simulations included all of the true causal variants in the genotype data set, and we were not relying on LD between markers and true causal genetic variants. Thus, the dilution sets were probably a good representation of the real data set compared to the marker sets that only included true causal variants.

Scenarios where h_f_^2^ was >0.1 showed greater power and robustness. This was particularly evident in the cluster causal model where power was over 0.7 for all dilution sets in scenarios with h_f_^2^ = 0.5 and h^2^ = 0.20 (Fig. 1). The only parameter for which the estimation deteriorated with increasing h^2^_f_ was the partitioning of genomic variance between the markers included in the genomic feature and the remaining markers for the dilution sets (estimated ${\hat{\mathrm{h}}}_{\mathrm{f}}^2$). At low dilution or low h^2^_f_, we achieved unbiased estimates of the proportions of genomic variance that could be attributed to the genomic feature (Fig. 4). However, at high h^2^_f_, the model overestimated the proportion of genomic variance that was attributed to the genomic feature in dilution sets. This overestimation was positively correlated with the number of non-causal markers included in the marker set.

#### Impact on predictive ability

In the h^2^ = 0.20 simulated data set, the predictive ability of the genome feature model was heavily influenced by dilution and h_f_^2^ (Fig. 5). When the dilution was minimal, the predictive ability of the GFBLUP model (equation (M_GF_)) was clearly improved compared to that of the standard GBLUP (equation (M_G_)) in most simulation scenarios. This result indicates that being able to separate the true causal variants from the non-causal variants in the GFBLUP would improve predictions, even in populations with relationship structures as tight as in the Danish Duroc breed. If we want to optimize the GFBLUP approach, it is critical to have enough power to correctly detect regions with causal markers in the training population. The use of data available from sources outside of the training data set could increase the ratio of causal variants to non-causal variants among the markers included in the genomic feature.

### Comparing genomic models using real data

Incorporating information about QTL-based genomic features in the prediction model increased prediction ability for LMP compared with the standard GBLUP model. For the two other traits, predictive ability was not improved by use of any GFBLUP approach. Selecting genomic features based on single markers or genomic blocks that showed significant effects in the training population produced GFBLUP models that explained a lot of the variance found in the training population. For many of the tested models, estimates of genomic heritability exceed the heritability in the data set containing all 34,425 boars (including non-genotyped animals), as well as the genomic heritability estimated using the standard GBLUP (Fig. 6). However, these models did not show greater prediction ability, suggesting data over-fitting. In other words, that some of the significant markers were not actually in linkage disequilibrium, LD, with true causal variants. In contrast, with the QTL set models, genomic heritability estimates were always in the same range as with the standard GBLUP. The main difference between the QTL sets and the two other genomic feature classes (single-marker and block sets) was that the QTL sets included data previously obtained from sources other than the training data, i.e*.* literature results. This additional information may have decreased the risk of including non-causal genomic regions or markers in **G**_f_. Additionally, although the QTL set significance was evaluated based on the same training set as the single-marker and block sets, some QTL sets included several marker blocks that were separated on the genome by substantial distance. This could have resulted in less weight being placed on spurious associations in the QTL sets. Results from the simulation study supported the interpretation that QTL set models included less non-causal genomic regions in **G**_f_ than the other genomic feature classes. Figure 4 shows that GFBULP models gave unbiased estimations of the proportion of genomic variance explained by ${\mathbf{G}}_{\mathrm{f}}\left({\hat{\mathrm{h}}}_f^2\right)$, provided dilution by non-causal variants was low. If **G**_f_ included higher proportions of non-causal variants the GFBLUP models attributed too much of the genetic variation to **G**_f_. The middle panel of Fig. 6 displays that ${\hat{\mathrm{h}}}_{\mathrm{f}}^2$ is close to 1 for all the GFBLUP models except the QTL set models, in agreement with what we would expect if **G**_f_ included a high proportion of markers that were not directly linked to causal variants in addition to markers that were linked to real causative genetic variation.

Our present approach is similar but not identical to the BLUP|GA method used by Zhang *et al.* [6]. In their study, they improved the accuracy of genomic prediction by weighing each SNP according to how often it has been associated with the investigated trait in the literature. In contrast, we first evaluated the association of all pig QTL sets with the investigated trait in the training population, partitioned the markers accordingly, and then estimated the variance components from the data. When we applied the BLUP|GA method to our dataset, the predictive ability and estimates of $\hat{{\mathrm{h}}^2}$ were similar to those found with the standard GBLUP model. Like GFBLUP, different Bayesian methods allow differentiation between markers depending on estimates of their genetic variance. However Bayesian lasso does not perform better than standard GBLUP on a subset of the data used in the current study [12], in addition Speed and Balding [7] found their Adaptive MultiBLUP model to perform as well or better than Bayesian sparse linear mixed models.

Considering the high relatedness of the animals in our data set, the 5.6 % increase in predictive ability compared to the standard GBLUP for LMP is not negligible. The predictive abilities of our models were lower than the previously reported reliabilities for ADG and FE in the same population [13]. This is because, in contrast to Christensen *et al.* [13], we left a one-year gap between our training and validation populations. Population structure has two major influences on genomic prediction. First, a normal GBLUP will perform well in populations with strong long-range linkage disequilibrium, although we tried to minimize this issue by leaving one generation between the training and the validation population. This means that the genomic relationship matrix will, at least to some degree, be correlated with any genetic variant that influences the trait that is being predicted [14]. Since the GBLUP model captures a substantial part of the additive genetic variance in highly structured populations, there is less scope for improvement. The second influence of population structure is that high long-range linkage disequilibrium makes it difficult to pinpoint markers that are close to the causal variants. These problems are common to many other genomic feature modelling approaches, including the Adaptive MultiBLUP method proposed by Speed and Balding [7]. They showed that partitioning markers into classes with distinct effect-size variances increased prediction ability for human diseases, but did not improve prediction of traits within a highly structured inbred mouse population.

Comparing results from the three traits revealed more significant QTL sets for LMP (Table 1), which was also the trait that displayed the highest estimated genomic heritability and predictive ability in all models. Additionally, compared to the two other traits, LMP showed a much lower increase in predictive ability upon inclusion of individuals from 2011 in the validation set (results not shown). There are several possible explanations for the lack of improved predictive ability by QTL set models for ADG and FE. The QTL data may not contain QTL regions that are related to these traits in our populations. However, this is unlikely, since ADG is one of the more intensively studied traits in pigs. A more likely explanation is that the genetic variation in these two traits may have been too low to allow accurate selection of QTL sets with the number of observations in our training population. This interpretation was supported by our re-evaluation of the QTL sets combining the training and validation populations (results not shown). We found that of the five QTL sets that were significant for FE at *p* < 0.05 (Table 1), only two were also significant in the new analysis including all individuals. Similarly, for ADG, only one of eight QTL sets was still significant in the new analysis. In contrast, for LMP, of the 11 QTL sets that were significant for LMP (listed in Table 1), 7 were significant when all individuals where included in the analysis. A third possibility is that the strong degree of relatedness within the Danish Duroc population [9] may have posed problems in terms of partitioning the genomic variance between **G**_**f**_ and **G**_**r**_.

The results from the simulation study show that the main factors determining whether prediction accuracy is increased by GFBLUP, compared to standard GBLUP, is the proportion of genetic variance that can be explained by the markers in **G**_f_, and the amount of dilution introduced by adding markers that are not linked to causal variants in **G**_f_. These findings suggest that the main explanation for the lack of improvement by the GFBLUP models in prediction ability for ADG and FE is lack of power to distinguish markers linked to causal genetic variation.

### QTL sets associated with growth phenotypes

Below, we discuss in greater detail the biology of the QTL sets that were included in **G**_**f**_ in the best-performing GFBLUP for LMP.

#### Muscle QTL sets

Lean meat percentage is a measure of the proportion of the pig’s body that comprises muscle tissue; thus, we expected that QTL sets for muscle traits would be among the most significant. The muscle-related QTL sets included in **G**_**f**_ of the best-performing GFBLUP included longissimus dorsi muscle thickness, type IIa muscle fibre quantity, skeletal muscle fibre quantity, and type IIb muscle fibre quantity. Within our data set, LMP seemed to be more explained by the QTL sets associated with numbers of fast muscle fibres (type II fibres) than by QTL sets associated with slow muscle fibres (type I muscle fibre quantity; *p* = 0.16) or fibre size (skeletal muscle fibre size trait; *p* = 0.67). Some studies find that increased meatiness is mainly influenced by increased fibre size and not number [15]; however, selection for increased leanness reportedly leads to increased type II muscle fibre proportions but not changes in fibre size [16].

#### Adipose QTL sets

The amount of fat deposited during growth can be lowered either by reducing the number of adipocytes or reducing the size of individual fat cells. Two of the included QTL sets were associated with fat traits: white adipocyte size trait, and white adipose amount. In Duroc boars, LMP seemed to be less impacted by the number of adipose cells than by their size (adipocyte quantity; *p* = 0.26).

#### Immune system QTL sets

Three QTLs included in **G**_f_ in the best-performing GFBLUP were tightly associated with immune function: leukocyte quantity, CD4-positive T cell quantity, and blood interleukin-10 amount. Leukocytes (i.e. white blood cells) are immune system cells that increase in quantity as part of the defence against pathogens. Therefore, a high leukocyte quantity is an indicator of infection. CD4-positive T cells are part of the adaptive immune system, and are involved in antibody expression. They also help activate and regulate the other lymphocytes, *e.g.* via production of the anti-inflammatory cytokine interleukin-10 [17, 18].

Linkage between LMP and the immune response could occur through several possible mechanisms. Strong activation of the immune system requires energy, and could divert resources that would otherwise be used for growth. High immune system activation can also lead to low protein:lipid ratios [19]. Additionally, the immune system plays an important role in influencing gut microbiota. In mammals, obesity is associated with an abnormal proportion of certain gram-positive bacteria [20]. Genes linked to the immune system are notoriously high in genetic variation due to pathogen-driven negative frequency-dependent selection for new alleles [21]. Thus, these genes could explain a significant proportion of the genetic variation purely by chance. Although the mechanism of involvement remains unclear, immune functions are an interesting avenue for research regarding factors affecting production traits.

#### Body conformation QTL sets

Several of the significant QTL sets were related to body conformation—namely, cannon bone circumference, head mass, testes mass, total foot mass, outer ear area, nipple quantity, vertebra quantity, and thoracic vertebra quantity. These body conformation traits might be indicators of the balance between lean meat and fat in the carcass composition, which is a major determinant of production traits in pigs [22].

## Conclusions

Our present simulation studies demonstrated that the GFBLUP model could have greater predictive ability than the standard GBLUP, provided that enough causal variants were included in the genomic feature to explain >10 % of the genomic variance, and that dilution by non-causal markers was minimal. Addition of results from literature clearly increased predictive ability. In the observed data set, we could increase predictive ability by including QTL-related data obtained outside of the training data set, but only for the trait with the highest heritability.

## Methods

### Observed data

Phenotypes for three traits were available from 34,425 pure-bred Duroc boars that were part of the Danish pig-breeding system. All boar testing was conducted at the national test station Bøgildgaard (Pig Research Centre, Danish Agriculture and Food Council, Denmark). The phenotypic records included average daily gain (ADG; g/day) from 30 kg–100 kg body weight, feed efficiency (FE; feed units/kg gain), and lean meat percentage (LMP). At the end of the test period, all boars were weighed and back-fat was measured by ultrasound and used to predict LMP. The pedigree was traced back to 1984, consisted of 419,961 animals, and included 256 unknown parents (base animals).

Genotypes were obtained for 3,085 of the phenotyped animals using either Illumina’s Porcine SNP60 BeadChip or Illumina’s 8.5 K GGP-Porcine Low Density Bead SNP chip. Genotypes of animals genotyped with the 8.5 K SNP chip were imputed to the SNP60 chip as described by [23]. A total of 33,029 of the 60 K SNPs fulfilled the following editing criteria and were used in our analyses: call rate of SNPs greater than 90 %, minor-allele frequency greater than 0.01, showed Hardy Weinberg expectations (*p*(*χ*_1_^2^) > 10^− 7^), and allocated a chromosomal position on build Sscrofa10.2 [24]. All animal samples had call rates greater than 80 %.

#### Adjusted phenotypes used in genomic model analyses

The phenotypes used in the genomic model analyses were derived from phenotypic records of growth traits adjusted for relevant environmental factors using the following linear mixed model:

$$ \mathbf{y}=\mathbf{X}\mathbf{b}+{\mathbf{Z}}_{\mathrm{p}}\mathbf{p}+{\mathbf{Z}}_l\mathbf{l}+{\mathbf{Z}}_{\mathrm{a}}\mathbf{a}+\mathbf{e}\kern3em \left({\mathrm{M}}_{\mathrm{a}}\right) $$

where **y** is a vector of phenotypic observations; **X** is a design matrix for the fixed effects (starting weight, year, and section); **Z**_p_ is a design matrix for the random effect of pen; **Z**_*l*_ is a design matrix for the random effect of litter; **Z**_a_ is a design matrix for the random additive genetic effect of animal (inter-individual variation determined from pedigree information); **b** is the vector of fixed effects; **p**, **l**, and **a** are vectors of random pen effects, litter effects, and animal effects, respectively; and **e** represents the residuals. The random effects and residuals were assumed to be independent normally distributed variables described as follows: **p** ~ N(0, **I**_p_σ_p_^2^), **l** ~ N(0, **I**_l_σ_l_^2^), **a** ~ N(0, **A**σ_a_^2^), and **e** ~ N(0, **I**σ_*e*_^2^). The relationship matrix **A** was constructed using pedigree information. The variance components σ_p_^2^, σ_l_^2^, σ_a_^2^, and σ_e_^2^ were estimated using an average information REML procedure [25]. The adjusted phenotypes used as response variables for genomic model analysis were calculated as the sum of the estimated residuals **e** and additive genetic effects **a**. This procedure enabled the use of all available phenotypes to estimate the fixed and random environmental effects, regardless of whether the animal was genotyped.

### Statistical analyses using genomic models

We performed analyses using two different genomic models: GBLUP and GFBLUP using prior information on genomic features. These models were compared based on their predictive abilities, the proportion of phenotypic variance explained by genomic effects, and the precision of the estimated genomic parameters. Analyses utilized both observed and simulated phenotypic data.

The GFBLUP model was based on a linear mixed model including two random genomic effects:

$$ \mathbf{y}\hbox{'}=\mu +\mathbf{Z}\mathbf{f}+\mathbf{Z}\mathbf{r}+\mathbf{e}\kern3em \left({\mathrm{M}}_{\mathrm{GF}}\right) $$

where **y** is the vector of adjusted phenotypes, ***µ*** is an overall mean, **Z** is the design matrix linking observations to genomic values, **f** is the vector of genomic values captured by genetic markers linked to the genomic feature of interest, ***r*** is the vector of genomic values captured by the remaining set of genetic markers, and ***e*** is the vector of residuals. The random genetic effects and the residuals were assumed to be independent normally distributed values described as follows: **f** ~ N(0, **G**_***f***_*σ*_*f*_^2^), **r** ~ N(0, **G**_***r***_*σ*_*r*_^2^), and **e** ~ N(0, **I***σ*_*e*_^2^).

The GBLUP model was based on a linear mixed model including only one random genomic effect:

$$ \mathbf{y}\hbox{'}=\mu +\mathbf{Zg}+\mathbf{e}\kern3em \left({\mathrm{M}}_{\mathrm{G}}\right) $$

where **y** is the vector of phenotypic observations, ***µ*** is an overall mean, **Z** is the design matrix linking observations to genomic values, **g** is the vector of genomic values captured by all genetic markers, and ***e*** is the vector of residuals. The random genomic values and the residuals were assumed to be independent normally distributed values described as follows: **g** ~ N(0, **G***σ*_*g*_^2^) and **e** ~ N(0, **I***σ*_*e*_^2^).

The additive genomic relationship matrix **G** was constructed using all genetic markers [2] as follows: **G** = **WW**^'^/m, where **W** is the centered and scaled genotype matrix, and m is the total number of markers. Each column vector of **W** was calculated as follows: ${\boldsymbol{w}}_{\boldsymbol{i}}=\frac{{\boldsymbol{m}}_{\boldsymbol{i}}-2{p}_i}{\sqrt{2{p}_i\left(1-{p}_i\right)}}$, where *p*_*i*_ is the minor allele frequency of the *i*^*th*^ genetic marker, and **m**_**i**_ is the *i*^*th*^ column vector of the allele count matrix **M**, which contains the genotypes coded as 0, 1, or 2 depending on the number of copies of the minor allele. The **G**_**f**_ and **G**_**r**_ was constructed similarly using only the genetic marker set defined by the genomic feature and the remaining set of markers, respectively.

#### Estimation of genomic parameters

The variance components ${\hat{\upsigma}}_{\mathrm{f}}^2,{\hat{\upsigma}}_{\mathrm{r}}^2,{\hat{\upsigma}}_{\mathrm{g}}^2,\mathrm{and}\ {\hat{\upsigma}}_{\mathrm{e}}^2$ were estimated using an average information REML procedure [25], as implemented in DMU [26]. For this process, we used the generalized inverse of the genomic relationship matrices. This was necessary because these matrices were not full rank due to centring, as well as in cases where the number of genetic markers was smaller than the number of phenotypic records. From these variance components, inferences on genomic heritability were based on the following ratios: ${\hat{h}}_{GBLUP}^2=\frac{{\hat{\upsigma}}_{\mathrm{g}}^2}{{\hat{\upsigma}}_{\mathrm{g}}^2+{\hat{\upsigma}}_{\mathrm{e}}^2}$ for GBLUP, and ${\hat{h}}_{GFBLUP}^2=\frac{{\hat{\upsigma}}_{\mathrm{f}}^2 + {\hat{\upsigma}}_{\mathrm{r}}^2}{{\hat{\upsigma}}_{\mathrm{f}}^2+{\hat{\upsigma}}_{\mathrm{r}}^2+{\hat{\upsigma}}_{\mathrm{e}}^2}$ for GFBLUP. Inferences on partitioning of genomic variance in GFBLUP were based on the following ratios: ${\hat{h}}_f^2=\frac{{\hat{\upsigma}}_{\mathrm{f}}^2}{{\hat{\upsigma}}_{\mathrm{f}}^2+{\hat{\upsigma}}_{\mathrm{r}}^2}$ and ${\hat{h}}_r^2=\frac{{\hat{\upsigma}}_{\mathrm{r}}^2}{{\hat{\upsigma}}_{\mathrm{f}}^2+{\hat{\upsigma}}_{\mathrm{r}}^2}$. These ratios quantified the proportions of total genomic variance explained by the genetic markers in the genomic feature, and by the remaining set of genetic markers not part of the genomic feature.

#### Model statistics for comparing genomic models

The predictive abilities of the models were assessed using bootstrap validations. The training population included 1,814 of the animals born in 1998–2010 and for which we had both phenotypes and genotypes. To ensure a gap of at least one generation from the training population, the validation population comprised 1,271 genotyped boars that were born between 2012 and 2014. We evaluated the models’ predictive abilities by calculating the correlation between the observed phenotype **y** and the total genomic value—which was $\hat{\mathbf{g}}$ for GBLUP, and $\hat{\mathbf{g}} = {\hat{\mathbf{g}}}_f + {\hat{\mathbf{g}}}_r$ for GFBLUP. This was completed by first randomly sampling 1/5 of the animals in the validation set, and then calculating the correlation between the observed phenotype and the total genomic value. This procedure was repeated 100 times and the predictive ability was defined as the average correlation of 100 bootstrap samples (± standard error).

### GBLUP approach for identifying genomic features associated with phenotypes

To identify phenotype-associated genomic features, we used a GBLUP-derived procedure for evaluating the collective action of a set of genetic markers. This approach is based on computing a summary statistic for the set of genetic markers that measures the degree of association between the genetic feature and the phenotypes. This summary statistics can be computed several ways using single-marker effects and test statistics.

#### Single-marker effects and test statistics

The single-marker effects $\hat{\mathbf{s}}$ can be computed from the predicted genomic effect $\hat{\mathbf{g}}$ [25, 27] as follows:

$$ \hat{\mathbf{s}}=\mathbf{W}\hbox{'}{\left(\mathbf{W}\mathbf{W}\hbox{'}\right)}^{-1}\hat{\mathbf{g}} $$

The variance of the single-marker effects can be calculated with the following equation:

$$ Var\left(\hat{\mathbf{s}}\right)=\mathbf{W}\hbox{'}{\left(\mathbf{W}{\mathbf{W}}^{\hbox{'}}\right)}^{-1}\mathrm{V}\mathrm{a}\mathrm{r}\left(\hat{\mathbf{g}}\right){\left(\mathbf{W}\mathbf{W}\hbox{'}\right)}^{-1}\mathbf{W}\hbox{'} $$

In this expression, $\mathrm{V}\mathrm{a}\mathrm{r}\left(\hat{\mathbf{g}}\right)$ is the variance of the predicted genomic effect [28], which can be derived from the inverse of the coefficient matrix of the mixed model equations as **G** − **C**^**gg**^, where **C**^**gg**^ corresponds to the genomic effects.

A test statistic for a single genetic marker effect is computed as follows:

$$ {t}_{{\hat{\boldsymbol{s}}}_{\boldsymbol{j}}}=\frac{{\hat{\boldsymbol{s}}}_{\boldsymbol{j}}}{\sqrt{Var\left({\hat{\boldsymbol{s}}}_{\boldsymbol{j}}\right)}} $$

where $Var\left({\hat{\boldsymbol{s}}}_{\boldsymbol{j}}\right)$ is the estimate of variance of the j’th element of $\hat{\mathbf{s}}$, obtained from the j’th element of the diagonal of the (co)variance matrix of the single-marker effects. Under the null hypothesis that ${\hat{\boldsymbol{s}}}_{\boldsymbol{j}} = 0$, it is assumed that ${t}_{{\hat{\boldsymbol{s}}}_{\boldsymbol{j}}}$ follows a t distribution with df_e_ residual degrees of freedom [29]. The residual degrees of freedom df_e_ is computed as tr(**I**–**H**), which is equivalent to n-tr(**H**) where n is the total number of phenotypic observations and tr(**H**) represents the degrees of freedom occupied by the penalised fit (*e.g.* the linear mixed model fit). The hat matrix **H** transforms **y** into $\hat{\mathbf{y}}$ [30]. Although the individual *p* values calculated using this method differ from those obtained via traditional methods, the ranking of the *p* values will be the same.

#### Summary statistic for a genomic feature derived from single-marker statistics

For each genomic feature, we constructed an appropriate summary statistic that measured the degree of association between the marker set and the phenotypes. We considered two different summary statistics. The first summary statistic was based on counting the genetic markers in the feature that were associated with the trait phenotype, as follows:

$$ {\mathrm{T}}_{\mathrm{count}} = {\displaystyle \sum_{i=1}^{m_f}}\mathrm{I}\left({t}_i>{t}_0\right) $$

where m_f_ is the number of markers in the feature, t_i_ is the i’th single-marker test statistic (*e.g*. t-statistic), t_0_ is an arbitrarily chosen threshold for the single-marker test statistics, and I is an indicator function that has a value of 1 if *t*_*i*_ > *t*_0_. However, no matter how the threshold is selected for determining “significant associations,” it is somewhat arbitrary, and genetic markers with slightly differing test statistics may be treated completely differently. By design, this test has high power to detect association if the genomic feature harbours genetic markers with large effects, but it will not detect a genomic feature with many genetic markers having small to moderate effects [31]. In such a case, it would be more powerful to use a summary statistic, such as the mean or sum of the test statistic for all genetic markers belonging to the same genomic feature. Thus, we also utilized a second summary statistic based on summing the single genetic marker test statistics in the feature, as follows:

$$ {\mathrm{T}}_{\mathrm{sum}}={\displaystyle \sum_{i=1}^{m_f}}{t}_i^2 $$

where t_i_ represents the i’th single variant test statistics, *e.g.* marker effects or t-statistics.

#### Testing for association between a genomic feature and a phenotype

A genomic feature was considered significant if the associated summary statistics were more extreme than the cut-off set based on an empirical distribution of random marker sets of same size as the genomic feature. This was tested using a competitive null hypothesis, i.e*.* that the degree of association of the feature set was the same as that of a random marker set [32]. To this end, we obtained an empirical distribution of the test statistic by sampling random marker sets. A null hypothesis is only competitive if the parameters influencing the summary statistic are identical to the alternative hypothesis. Thus, there must be an equal number of markers for the random set and the true set, and the correlation structure among markers (due to linkage disequilibrium) should be retained. The empirical distribution of the summary statistics was obtained using the following permutation procedure. First, the observed test statistic was ordered accordingly to the physical position of the SNPs, and an element (i.e*.* one test statistic) was randomly selected from this vector. All elements were then shifted to new positions—such that the selected one became the first element, with the remaining SNPs shifted to new positions, but maintaining the original order. A new summary statistic was then computed based on the original position of the genomic features. This uncouples any associations between SNPs and the genomic feature, while retaining the correlation structure among test statistics. The permutation was repeated 1,000 times for each set in the feature class, and empirical *p* values were obtained through one-tailed tests of the proportion of randomly sampled summary statistics larger than that observed.

#### Genomic feature classes

Several strategies were used to define genetic marker sets that formed different classes of genomic features used in GBLUP and GFBLUP model analyses.

First, genomic features were derived from single-marker association test statistics (single-marker sets). A standard *t*-test was used to assess the single-marker statistical significance of the regression effect for individual SNPs. When an SNP was determined to be significantly associated with the genomic value based on a pre-specified significance cut-off level, the corresponding genome regions were then considered to define a “genomic feature.” These steps were repeated with decreasing significance cut-offs, thereby increasing the genomic region of the feature (SNP set).

Second, including or excluding SNPs from a genomic feature based on single-marker association tests can result in over-fitting of the data [33]. To ameliorate this risk, we created block sets of 50 markers that were physical adjacent on the genome, and we tested the associations of these marker sets with the trait using the above-described summary statistics. The significance of the association between the marker sets and the trait was determined using a pre-defined set of cut-off levels. Marker sets with *p* values below the cut-off were included in the genomic feature set.

Third, to assess the benefit of including prior data in GFBLUP models, we derived genomic features from the summary statistics of a group of genetic markers defined by a previously identified QTL region (a QTL set). The QTLs recorded in the Pig QTL database [11] are organized based on trait ontology, and we used the 167 traits listed in the Vertebrate Trait Ontology column. A trait can have multiple associated QTLs originating from several sources. We utilized the QTLs comprising the QTL set for the selected trait. The markers of our data set were grouped according to the genomic locations of QTL sets for the 167 trait categories downloaded from the database. The genomic region spanned by each individual QTL was standardized to 250 kb on each side of the QTL midpoint. Only QTL sets spanning >2 SNPs were used in the analysis. A marker set containing the SNPs that was not included in any of the QTL sets and a set containing all markers was added to this genomic feature class, resulting in a total of 169 tested marker sets. The number of SNPs in each QTL set is shown in Additional file 2.

### Simulated data

We also established a series of simulation studies to investigate factors influencing the power to detect genomic features affecting the trait phenotype, estimation of genomic parameters, and prediction ability of the two tested linear mixed models. We used the method described in [34] pp. 98. The genetic values and residuals were simulated in R using the function mvrnorm from the library MASS [35]. The factors varied in the simulations included genomic heritability (*h*^2^), proportion of genomic variance explained by causal SNPs in the genomic feature (*h*_*f*_^2^), proportion of non-causal SNPs in the genetic marker set defined by the genomic feature (*dilution*), genome distribution of causal SNPs (*causal model*) (i.e*.* how the causal SNPs were physically distributed on the genome: random or clustered), and the number of phenotypic observations available for analysis (N_obs_).

#### Genotypes

The simulations were based on the real genotype data set including 3,085 individuals and 33,029 SNPs. In all scenarios, the number of causal SNPs was equal to 1,000. Causal sets were divided into two subsets. The first subset *C*_1_ included 100 SNPs and was used as the causal SNP set in the genomic feature that explains 10 %, 20 %, 30 %, or 50 % of the genomic variance. The second subset *C*_2_ included 900 SNPs and explained the remaining genomic variance. To mimic relevant genetic scenarios, the genome distribution of the causal SNPs in the genomic feature was simulated using two different causal models: a *random* and a *cluster* model. The cluster model illustrated causal SNPs among connected genes in QTL regions. On the other hand, the random model provides an example of a trait with causal variants distributed in genes, which are linked to many different processes such that the pattern seems *random*. For the clustered causal model, the 100 causal SNPs in *C*_1_ were chosen from 20 randomly selected genomic regions spanning 50 SNPs each, and the remaining 900 SNPs in *C*_2_ were randomly selected from the complete SNP set. For the random causal model, the SNPs in *C*_1_ and *C*_2_ were randomly selected from the complete SNP set. To investigate the effects of non-causal SNPs within the causal sets, we added an increasing number of non-causal SNPs (100, 200, …, 1,900, 2,000), to the causal sets, in a process referred to as *dilution*. To determine the false-positive rate, 50 marker sets (referred to as a non-causal SNP set) of varying sizes (100, 500, 1,000, and 5,000) were sampled among the non-causal SNPs.

#### Phenotypes

Phenotypes were simulated using the following linear model: **y** = **g**_1_ + **g**_2_ + **e**, where g_1_ ~ N(0, **G**_1_ * *σ*_*g*1_^2^), g_2_ ~ N(0, **G**_2_ * *σ*_*g*2_^2^), and e ~ N(0, I * *σ*_*e*_^2^). G_1_ and G_2_ are the genomic relationship matrices for causal SNPs in C_1_ and C_2_, respectively. The total phenotypic variance *σ*_P_^2^ = *σ*_g1_^2^ + *σ*_g2_^2^ + *σ*_e_^2^ was 100 in all scenarios. We simulated data under additive genomic heritabilities $\left({h}^2=\frac{\sigma_{g1}^2+{\sigma}_{g2}^2}{\sigma_{g1}^2+{\sigma}_{g2}^2+{\sigma}_e^2}\right)$ of 0.1, 0.2, or 0.3, to analyse scenarios with low to intermediate heritabilities, reflecting those observed in the real data. To analyse scenarios with non-uniform SNP effects, the proportion of additive genomic variance explained by the causal SNPs in *C*_1_$\left({h}_f^2=\frac{\sigma_{g1}^2}{\sigma_{g1}^2+{\sigma}_{g2}^2}\right)$ was varied across scenarios: 0.1, 0.2, 0.3, or 0.5. These parameters were investigated for three population sizes (N_obs_): 1000 (1 K), 2000 (2 K), and 3000 (3 K). These variations resulted in a total of 72 individual simulated data sets [3 (N_obs_) × 3 (h^2^) × 4(h_f_^2^) × 2 (causal model)], which were each replicated 50 times. Table 2 presents an overview of the factors included in the simulation. The simulated data were analysed using the above-described linear mixed models, permutation, and cross validation procedures.

### Ethics

The present study was not subject to ethical approval since it was based on pre-existing data belonging to the Danish Agriculture and Food Council, Pig Research Centre, and did not require the application of additional experimental procedures. The simulated data is available upon request.

### Availability of data and materials

The genotypic and phenotypic data on the Danish Duroc population used in this study is private property of the Danish pig breeders and the authors are not at liberty to disclose them in the public domain. However, the simulated data are available upon request.

## Additional files

Additional file 1:
**Figure depicting the power to detect the genomic feature marker set using either the sum of squared marker effects (Sum B2), the sum of squared t-statistics (Sum T2), with a threshold of 0.01 (Cnt1), or with a threshold of 0.05 (Cnt5).** (DOCX 42 kb) Additional file 2:
**The QTLs recorded in the Pig QTL database [** 11 **] are organized based on trait ontology, and we used the 167 traits listed in the Vertebrate Trait Ontology column.** A marker set containing the SNPs that was not included in any of the QTL sets and a set containing all markers was added to this genomic feature class, resulting in a total of 169 tested marker sets. The number of SNPs associated with each of the 169 QTL sets is shown in Additional file 2 (XLSX 48 kb)

## Abbreviations

GFBLUP: Genomic feature best linear unbiased prediction
h_*f*_^2^: Proportion of genomic variance caused by the causal markers in the genomic feature set
C_1_: Causal markers in the genomic feature marker set
C_2_: Causal markers not in the genomic feature marker set.
