# Supplementary material for: Increased prediction accuracy using a genomic feature model including prior information on quantitative trait locus regions in purebred Danish Duroc pigs
Source: BMC Genet. 2016 Jan 5;17:11. doi: 10.1186/s12863-015-0322-9 (PMC4700613; doi:10.1186/s12863-015-0322-9)
Supplement: Additional file 1: — Figure depicting the power to detect the genomic feature marker set using either the sum of squared marker effects (Sum B2), the sum of squared t-statistics (Sum T2), with a threshold of 0.01 (Cnt1), or with a threshold of 0.05 (Cnt5). (DOCX 42 kb) [file 12863_2015_322_MOESM1_ESM.docx]

**Additional file 1**

Figure depicting the power to detect the genomic feature marker set using either the sum of squared marker effects (Sum B2), the sum of squared t-statistics (Sum T2), $T_{\mathrm{count}}$ with a threshold of 0.01 (Cnt1), or$T_{\mathrm{count}}$ with a threshold of 0.05 (Cnt5). In all scenarios, the sum of the squared value of the single-marker t-test statistic (t^2^) of the markers in the genomic feature performed as well as or better than the other single-marker test statistics.
